# Developing intercultural competence through a cultural metacognition-featured instructional design in English as a foreign language classrooms

**DOI:** 10.3389/fpsyg.2023.1126141

**Published:** 2023-02-09

**Authors:** Lin Huang

**Affiliations:** School of Foreign Languages, Xinyang Agriculture and Forestry University, Xinyang, China

**Keywords:** intercultural competence, cultural metacognition, English as a foreign language, instructional design, intercultural education

## Abstract

The soaring demand for intercultural competence (IC) in the globalized world has made it a key concern in foreign language education. Most existing training on IC has often focused on providing immersive intercultural experiences, equipping learners with cultural knowledge, and simulating intercultural situations. However, some of these approaches may not be feasible in English as a foreign language (EFL) classrooms, nor are they effective to prepare learners to cope with the complexities and uncertainties in novel intercultural situations unless there specifically involves higher-order thinking. Thus, this study took a perspective of cultural metacognition and examined whether and how could an instructional design that highlighted cultural metacognition facilitate learners’ IC development in an EFL classroom at the tertiary level in Chinese mainland. Fifty-eight undergraduate students enrolled in an English Listening, Viewing, and Speaking course were involved in the instruction, and questionnaires and focus groups were employed for the data collection. A paired sample *t*-test revealed that there was a significant enhancement in students’ intercultural competence in terms of affective, metacognitive, and behavioral dimensions, but not in the knowledge dimension. Thematic analysis indicated that the instructional design was effective in supporting students’ intentional knowledge acquiring, developing positive intercultural attitudes, and promoting the translation of cognition into actions. The findings thus confirmed that the instructional design featuring cultural metacognition can be used in domestic EFL contexts, such as College English classrooms at the tertiary level in Chinese mainland, as an effective way of enhancing learners’ IC. This study also offered additional evidence of how students’ IC development was achieved through a range of metacognitive processes, which may provide implications for teachers to design their IC instructions in similar EFL educational settings.

## 1. Introduction

With globalization accelerating and labor mobility increasing, intercultural competence (IC) has been widely recognized as a necessary asset for individuals to communicate appropriately and effectively across cultural borders (Feng, 2009; Spitzberg and Changnon, 2009; Zhang and Zhou, 2019). IC is also seen as one of the core 21st-century competencies, a key to employability, and an essential life-long competence (Deardorff, 2015; Griffith et al., 2016). This trend exercises ever-rising influence and pressure on all levels of education, especially in higher education where students are in relatively high demand for intercultural abilities to compete in the increasingly international workplace (Gu and Zhao, 2021). Quite often, foreign language education is expected to bear the responsibility of equipping learners with the capabilities to navigate intercultural communication effectively in one or more foreign languages (McConachy and Liddicoat, 2016). In most tertiary institutions in Chinese mainland, intercultural education is integrated into English as a foreign language (EFL) courses for both English majors and non-English majors as English is widely recognized as a global *lingua franca* (Wang and Kulich, 2015; Fang and Baker, 2018). Compared with most IC training designs focusing on providing immersive intercultural experiences, such as study abroad programs and intergroup learning with culturally diverse peers (Peng et al., 2015; Wang and Kulich, 2015), one critical challenge for intercultural education in Chinese domestic EFL context, especially in the post-Covid era, is that learners can hardly engage in concrete and authentic interactions with people from distinct cultural backgrounds due to geographic, political, or economic constraints (Zhao and Coombs, 2011); Wang and Kulich, 2015).

To develop alternatives, classroom-based instructions on developing learners’ IC have been explored in EFL context. The most common approach is cognitive training which focuses on the transfer of cultural knowledge by using multimodel materials, such as reading texts, pictures, and videos (e.g., Ogan et al., 2009; Rodríguez and Carranza, 2017; Barrett, 2018; Wang et al., 2020). This approach is effective in enhancing learners’ cultural knowledge acquisition about certain countries, but it is impractical to expect learners to acquire everything about a specific culture. In addition, such country-specific knowledge cannot be transferred across cultural domains (Mor et al., 2013). Moreover, mere exposure to learning materials that contain cultural differences and intercultural communication rules like “do”s and “do not”s makes learners rely heavily on rote learning without any further development of higher-order thinking (Lane, 2009). Just as Tan and Chua (2003, p. 223) note, “Unless you know everything, what you need is thinking.” The possession of cultural knowledge alone may not be enough to lead to an enhanced outcome, rather cultural metacognition would be necessary to improve the understanding and use of cultural knowledge (Chua and Ng, 2017; Sharma, 2019). Another strand of research on intercultural teaching focuses on simulating authentic intercultural situations by using teaching strategies such as critical incidents, role-play, and task-based activities. However, such activities may limit learners’ IC to surface-level simulations unless these activities specifically involve metacognitive strategies (Loewenstein et al., 2003; Earley and Peterson, 2004). In other words, overemphasizing specific intercultural examples at the expense of developing cultural metacognition may fail to prepare learners to cope with complexity and uncertainty in novel settings (Earley and Peterson, 2004).

Metacognition, defined as “thinking about one’s own thoughts” (Hacker et al., 1998), involves the conscious and intentional monitoring and regulation of one’s acquired knowledge about cognitive issues and affective and cognitive activities (Flavell, 1987). Earley et al. (2006) extend the general idea of metacognition to the intercultural domain and put forward cultural metacognition, referring to individuals’ cultural consciousness and awareness when interacting with others from different cultural backgrounds. Cultural metacognition characterizes a higher-order mental process and goes further than the mere cognitive process of acquiring and understanding cultural knowledge (Sharma, 2019). Moreover, it reflects individuals’ awareness and willingness to understand, interpret and adjust their cultural knowledge and cognitive schema (Bogilović et al., 2017; Chin et al., 2022). Substantial evidence supporting the importance of cultural metacognition in intercultural learning can be found in the domain of cross-cultural psychology (e.g., Earley and Peterson, 2004; Ang et al., 2006; Earley et al., 2006; Klafehn and Banerjee, 2008; Leung et al., 2013; Van der Horst and Albertyn, 2018; Morris et al., 2019). Cultural metacognition is also referred to as a “new frontier in cross-cultural competence research” (Chiu et al., 2013, p. 846) and a “key to cross-cultural knowing” (Chin et al., 2022). However, so far very few studies integrate cultural metacognition in the design of IC instructions in a systematic way, and research on the potential contributions that this can make to learners’ IC development in EFL classrooms is even scarcer. Therefore, this study seeks to address such shortcomings by conducting a multistage instructional design that specifically addresses cultural metacognition in EFL classrooms at the tertiary level in Chinese mainland. Because of the novelty of this instructional design, this study combines a set of teaching techniques that have been proved efficient in promoting cultural metacognition. Furthermore, by using a mixed methods approach to examine its effects on learners’ IC development, this study hopes to advance understanding of whether and how students develop their IC by the facilitation of cultural metacognition.

## 2. Review of literature

### 2.1. Exploring a model for IC development

Terminologies and definitions of IC are not consistently used throughout the literature. There are several synonyms used to depict IC, including cross-cultural communication competence (Ruben, 1989), intercultural communicative competence (Byram, 1997), intercultural sensitivity (Bennett, 1998), cultural intelligence (Earley et al., 2006), global leadership competency (Bird et al., 2010), etc. Nonetheless, IC seems to be an encompassing label and a generally preferred term to describe complex capabilities of interacting effectively and appropriately with people from different linguistic and cultural backgrounds (Fantini, 2009; Ang et al., 2015; Dypedahl, 2018).

To identify what it takes to be interculturally competent, a range of IC models have been developed over the years (see detailed review by Spitzberg and Changnon, 2009; Leung et al., 2013; Griffith et al., 2016). The most widely recognized and cited model of IC in formal foreign language teaching settings is proposed by Byram (1997). This model displays a set of individual knowledge, attitudes and skills that are essential for a competent intercultural speaker. Compared to the framework of “competence” in general educational theory, Sercu (2004) argues that Byram (1997)’s IC model is effective in addressing domain-specific knowledge, cognitive strategies, and affective characteristics but insufficiently addresses the metacognitive dimension. Working as the secondary thoughts, metacognition can amplify, attenuate, or even reverse primary cognition (Petty et al., 2007), and it may lead to changes in feelings and behaviors, making it critical in developing individuals’ competence (Metcalfe and Finn, 2008). In intercultural education, for true IC to be achieved, one should at least have a high level of self-awareness, an enhanced ability to self-assess, and a proclivity to reflect on one’s experiences (Lane, 2009). These competencies all relate to metacognitive growth. Thus, an IC model that clearly identifies cultural metacognition as an integral component seems justified.

A process-oriented model of IC proposed by Deardorff (2006) is the one that informs the current study. This model is used to explain the ongoing process of IC development in higher education context and assumes that the entire cycle of IC development begins with intercultural attitudes and then, motivated by which, individuals further develop intercultural knowledge and skills. The desired internal outcome (metacognition) later might be achieved, after which external outcome (intercultural communication and behaviors) can be realized through interactions. The external outcome can then be fed into attitudes, making the cycle fulfilled. Although it is possible for individuals to achieve minimal external outcomes by having the foundational attitudes alone, adding knowledge, skills and metacognition could facilitate more effective and appropriate intercultural interactions (Deardorff, 2012). This model supports the effectiveness of explicit IC instruction in language classrooms (Huang, 2021), but at the same time, it recognizes that IC development does not just happen through learning about cultural knowledge and practicing related skills. Just as Chua and Ng (2017), and Sharma (2019) suggested, learning how to think intercultural is more important than acquiring actual knowledge. Therefore, under the guidance of this framework, this study emphasizes the developmental elements contributing to IC, especially the role of metacognition, such as empathy, flexibility, and ethnorelative thinking (Leung et al., 2013), in providing a process for behavioral outcomes. This study also follows its view that IC development is a dynamic and cyclical process rather than an end result.

### 2.2. Significance of cultural metacognition to IC development

As stated in the introduction, cultural metacognition plays a critical role in the development of IC, which is primarily achieved by facilitating psychological and behavioral outcomes (Lane, 2009; Chua et al., 2012; Leung et al., 2013). It is suggested that individuals with higher levels of cultural metacognition are more aware of how their own culture influences their behavior and interpretations of intercultural situations, and thus making them more likely to query cultural assumptions and regulate their mental models during and after the interactions (Brislin et al., 2006; Triandis, 2006). Cultural metacognition also correlates positively with mental processes, such as conscientiousness (Earley et al., 2006), contextualized thinking and cognitive flexibility (Klafehn and Banerjee, 2008), affective closeness (Chua et al., 2012), stereotype judgments (Yzerbyt and Demoulin, 2012), and expectations of cooperative and relationship-oriented goals (Mor et al., 2013), which further contribute to cooperative behaviors in intercultural interactions (Chua et al., 2012; Morris et al., 2019). In addition to intercultural collaborative behaviors, large-scale quantitative studies show that cultural metacognition predicts a number of other performance outcomes, such as individuals’ behavioral enactment conforming to the role expectations in tasks (Ang et al., 2006; Malek and Budhwar, 2013), behavioral adaptations to cultural differences (Ang et al., 2006; Li, 2020), and cultural decision making (Rockstuhl and Van Dyne, 2018). From the above review, it is possible and practical to predict that instructional designs specifically address cultural metacognition may provide a robust foundation for developing learners’ IC.

As for how cultural metacognition contributes to intercultural effectiveness, Van Dyne et al. (2012) identify three self-regulated mental processes that work throughout intercultural interactions, namely planning, checking, and awareness. More specifically, before a culturally diverse encounter, planning prompts individuals to anticipate what they need to do and develop specific action plans in order to achieve certain short-term or long-term objectives (Van Dyne et al., 2012). During and after interactions, checking involves reviewing and questioning assumptions and adapting mental models when actual experiences differ from expectations. Awareness should always be present as it is about the real-time consciousness of how cultural factors influence the mental processes and behaviors of self and others. Together, these three constituent elements of cultural metacognition actively contribute to the transformation of thoughts into interculturally appropriate actions (Van der Horst and Albertyn, 2018). However, the challenge remains how to engage students in such metacognitive processes.

It is found that learners do not engage in metacognitive processes automatically unless they are deliberately asked to participate in purposefully designed activities (Bransford et al., 1999; Bannert et al., 2015). Thus, it is critical to integrate cultural metacognition in intercultural instructions and encourage learners to apply metacognitive skills to learning. Extant studies (e.g., Kramarski and Mevarech, 2003; Lane, 2009; Bryce et al., 2015; Bae and Kwon, 2019) show that metacognitive skills are “teachable” (Bae and Kwon, 2019, p. 4). For example, explicit coaching or discussion about metacognitive skills is an effective way to show students why metacognition matters and when and how to use the skills (Bae and Kwon, 2019; Santangelo et al., 2021). Turning to intercultural education, such metacognitive skills may include self-monitoring, which is used to constantly check for one’s cultural understanding (Phelan, 2019; Liao and Thomas, 2020), regulating, which involves adapting cognitive activities and ensuring the achievement of cognitive goals (Liao and Thomas, 2020), perspective-taking, which is also a reflective skill and involves considering how cultural factors shape individuals’ thoughts and behaviors (Lee et al., 2013; Van der Horst and Albertyn, 2018), mindfulness, which helps individuals avoid inappropriate or automatic behaviors by learning to observe their own thoughts, emotions, and habits (Thomas, 2006; Van der Horst and Albertyn, 2018), etc. While Earley and Peterson (2004) suggested decades ago that a specific discussion of metacognitive strategies in intercultural training could promote learners’ ability to transfer a concept from an example case to a novel situation, explicit coaching or discussion on these cultural metacognitive skills has rarely been conducted in classroom instructions for enhancing IC. Some other practical techniques, such as reflective questions (Kramarski and Mevarech, 2003; Nunaki et al., 2019), and reflective writing (O’Loughlin and Griffith, 2020), have also been verified as efficient in enhancing metacognition, but have not been sufficiently explored in the context of intercultural learning. As the above review indicates, explicit instruction and reflective practices are two major ways of promoting cultural metacognition, but the idea that these teaching techniques are related to students’ IC development needs further empirical work. Therefore, there are good reasons to purposefully design an instruction that integrates these techniques and examine whether and how such intervention could engage students in the metacognitive processes and further promote students’ IC. Accordingly, two research questions were raised:

1. Is the instructional design that features cultural metacognition effective in facilitating EFL students’ IC development?
2. If yes, how does cultural metacognition contribute to EFL students’ IC development?

## 3. Methodology

### 3.1. Research setting and participants

To explore whether and how an instructional design focusing on cultural metacognition could facilitate students’ IC development in EFL context, this study was situated in an English Listening, Viewing, and Speaking course offered to Business English sophomores at an undergraduate university in Chinese mainland. The reason to focus on this course is for its clear teaching objective of IC development and its target students. As prescribed in the course syllabus, this course aims at enhancing students’ English listening skills, speaking skills, and intercultural abilities so that they can use English to communicate appropriately and effectively in both business and interpersonal settings. This course was taught by the author, a non-native EFL teacher, for 90 min each week. In a 16-week course design, 4 business-related topics were covered, namely making business appointments, meeting clients, entertaining clients, and visiting factories.

Fifty eight business English majors enrolled in the course as a compulsory subject in the third academic semester. They were selected as participants because there is a high potential for business-related majors to meet culturally diverse customers in the future workplace. Furthermore, as shown in Table 1, the participants were on average 19.3 years old and 87.9% were female. They all belong to the Han ethnic group (the largest ethnic group in the Chinese mainland, which takes up 91.11% of the population) (National Bureau of Statistics, 2021), and their native language is Chinese. None of them had international travel experience, but 25.9% of them had experience interacting with people from other cultures in English. Before implementing the instructional design, students were told the purpose of this study and were asked to sign the participant consent form voluntarily. Pseudonyms are used for all participants.

**Table 1:** Demographic information of the participants.

Variables	Numbers (%)
Educational level	
Undergraduate - sophomore	58 (100)
Gender	
Female	51 (87.9)
Male	7 (12.1)
Age	
18–19	36 (62.1)
20–21	21 (36.2)
22–23	1 (1.7)
Experiences of interacting with people from other cultures in English	
None	43 (74.1)
Yes	15 (25.9)
Experiences of international travel	
None	58 (100)
Member of ethnical minority in China	
No	58 (100)

### 3.2. An instructional design featuring cultural metacognition

In order to engage students in metacognitive processes, they were given a multistage instruction for 8 weeks, which covers two topics (i.e., meeting clients, and entertaining clients). The instructional lessons were structured following Deardorff’s (2006) process-oriented IC model. More specifically, the 8 lessons progressively covered the cyclical process starting from attitudes, knowledge and comprehension, skills, internal outcomes (metacognition), to external outcomes (intercultural communication and behaviors). Explicit coaching and discussion on metacognitive skills, and reflective questions and writing were the instructional strategies used to promote students’ cultural metacognition. The details of the lessons are shown in Table 2.

**Table 2:** Procedures of the lessons.

Lesson	Topic	Instructional design	IC targeted
Lesson 1&2	Meeting clients	Listening and viewing activities	Attitudes, knowledge, skills
Lesson 3		Explicit instruction on metacognitive skills Introduction to the Autobiography task	Cultural metacognition
Lesson 4		Group presentation of the Autobiography Teacher and peer feedback After-class reflective writing	Behaviors
Lesson 5&6	Entertaining clients	Listening and viewing activities	Attitudes, knowledge, skills
Lesson 7		Discussion and reflection on the use of metacognitive skills Introduction to the role-play task	Cultural metacognition
Lesson 8		Group presentation of role play Teacher and peer feedback After-class reflective writing	Behaviors

In the first two lessons, around the topic of meeting clients, cultural differences regarding verbal and non-verbal communication in the workplace were introduced through audio and video materials in the textbook. In addition to guiding students to do relevant listening exercises, the teacher drew students’ attention to cultural knowledge and trained their skills of listening, observing, analyzing, and interpreting the intercultural aspects embedded in the materials. These activities also aim at advancing students’ knowledge of cultural knowledge as well as cultivating their attitudes toward cultural differences, such as curiosity, respect, and openness.

In the third lesson, the teacher explicitly lectured students on cultural metacognitive skills including self-monitoring, regulating, perspective-taking, and mindfulness. The teacher then introduced a task which was inspired by “Images of Others – An Autobiography of Intercultural Encounters through Visual Media” (hereinafter abbreviated as the Autobiography), which is a tool published by the Council of Europe to develop learners’ IC by encouraging learners to explore the intercultural encounters through images represented in books, television, films, Internet, etc. (Byram et al., 2009). Considering that in-person intercultural experiences are not so common for students in domestic educational contexts, visual media provide important opportunities for them to engage with different cultures. Thus, the Autobiography can help students reflect on how they think and feel about a particular image and what they can learn from it for future intercultural encounters. In this task, students were encouraged to work in groups of 5–6 and choose a specific image that shows someone from different cultural background. A list of guiding questions adapted from the Autobiography was provided to elicit students’ perceptions and feelings of people from other cultures. Furthermore, to involve students in cultural metacognitive processes and promote them to examine themselves objectively, some questions were developed to address the three specific metacognitive mental processes (see examples in Table 3).

**Table 3:** Examples of guiding questions that address cultural metacognition.

Guiding questions	Metacognitive mental processes covered
If you were to actually meet the people shown in the image, what would you do or say? What do you think you could do to make it easier for you to understand each other?	Planning
How did you feel when you first saw the image? What you think caused your feelings? Thinking about the people in the image and yourself, what do you think are the main differences between them and yourself? What do you think caused those differences?	Checking
Do you think the people shown in the image would be pleased with this image of themselves? Do you think that the image is a stereotype of the people shown in the image?	Awareness

In lesson 4, students were asked to present the results of their group discussions in class. After each group’s presentation, other groups were encouraged to ask questions and discuss differences in their views. The teacher also put forward further questions either to encourage positive intercultural attitudes or challenge possible stereotypes and prejudices. Through receiving feedback and defending their own positions, students could continuously question their previous assumptions and adjust mental models. At the end of this lesson, students were given an after-class reflective written task that prompted them to reflect on their intercultural experiences in group work. This task included questions such as what helped you understand the people in the image? What do you think you can do to make it easier for you to understand each other? Has analyzing the image changed your thinking in any way? Will you do something as a result of analyzing the image?

Lesson 5 and 6 were delivered in the same way as the first two lessons but covered another topic, which is about cultural differences in business etiquette regarding entertaining clients. In lesson 7, the teacher first guided students to reflect on and discuss the metacognitive skills they used in the Autobiography task and further explained how these skills helped students in their intercultural experiences. Then, the teacher presented a task that requires students to role-play the following situation: suppose you are going to receive people shown in the image as your clients and you are planning some activities that can entertain and impress them, try to ask them as many appropriate questions as possible and discuss with them the schedule regarding sightseeing and dining in one city in China. In lesson 8, students were required to present their role-play dialogs in class. Other groups were encouraged to share comments on the presenters’ performances. At the end of this lesson, students were given an after-class reflective written task prompting them to reflect on their role play. Reflective questions include: what helped make you more appropriate and effective in the role-play? What would you do to further develop your intercultural competence?

### 3.3. Data collection and analysis

Taking account of the complex nature of IC, researchers (e.g., Fantini, 2009; Deardorff, 2015) highlight the necessity of using a multi-method, multi-perspective approach to assess IC. Hence, a mixed methods approach with self-reflective questionnaires and focus groups was employed to address the two research questions and triangulate the results.

Pretest and post-test questionnaires using a 6-point Likert scale (0 = strongly disagree and 5 = strongly agree) were administered to participants to see if the instructional design was effective in enhancing their IC. The questionnaire contains 32 questions in total. In addition to the 5 questions which addressed participants’ demographic information, 27 questions covering 4 subscales of IC, namely, knowledge, affective orientation, behavioral performance, and cultural metacognition were adapted from Chao’s (2014) Intercultural Competence Scale (ICS; Chao, 2014). Different from a number of existing self-assessment instruments that are intended for English as a second language (ESL) learners or learners who study overseas, ICS is specifically designed for EFL learners in higher education and written in Chinese. Hence, ICS can well address the population in this study and serve the research purpose. Also, the dimensions of IC covered in this questionnaire are consistent with Deardorff’s (2006) categorization of IC, and the detailed information is shown in Table 4. Fifty-eight students completed both the pretest and post-test before and after the instructional lessons. The data gained from questionnaires were analyzed by a paired sample t-test by SPSS 28.0 to determine whether there were statistically significant changes in students’ self-perceived IC after the instruction.

**Table 4:** Reliability and descriptive statistics of the questionnaire.

Dimension	Sub-dimension	N of item	Reliability (Cronbach’s α)		Mean		S.D.	
			Pretest	Post-test	Pretest	Post-test	Pretest	Post-test
Knowledge	Culture-general	9	0.91	0.93	27.03	27.66	6.03	6.18
	Culture-hybrid							
	Culture-specific							
Affective orientation	Motivation	6	0.90	0.87	14.19	17.21	4.28	5.20
	Willingness							
	Attitudes							
Behavioral performance	EFL abilities	7	0.92	0.87	19.43	21.21	5.03	5.38
	Communication strategies							
	Interactive behavior							
Cultural metacognition	Planning	5	0.89	0.86	11.71	15.12	3.02	4.48
	Self-monitoring							
	Reflecting							

According to Deardorff’s (2006) model, IC development is a process rather than an end result. Therefore, there is a need to explore the process of how learners develop their IC with the facilitation of the instructional design. In this study, the focus group, a qualitative method which is productive in gathering detailed opinions and knowledge around a particular topic (Cohen et al., 2018), was applied to elicit EFL learners’ perceptions of how the explicit coaching and discussion on cultural metacognitive skills, and reflective questions and writing help them develop intercultural knowledge, affect, and behavior. As the author taught the course and was familiar with the participants, the author held focus group interviews for each group upon the completion of the lessons. Fifty-four students voluntarily participated in the focus group interviews, and thus, 10 focus groups were conducted (*n* = 6, 6, 5, 6, 4, 5, 5, 6, 6, 5). To promote participation, the interviews were carried out in Chinese. The data derived from the focus group were transcribed verbatim and then analyzed following a thematic analysis which can be referred to Braun and Clarke (2006) guidelines. The author first read the transcripts iteratively and gain familiarization in order to generate initial codes. Through coding, all raw data were organized into meaningful groups (Braun and Clarke, 2006). Then, some codes were combined to form overarching main themes, and some codes were sorted into sub-themes (Braun and Clarke, 2006). A colleague of the author was invited to recheck the codes and themes to ensure that the data had been interpreted appropriately. To further achieve validity and credibility of the analysis, the codes were presented to 5 participants to check if there were any misinterpretations (Dörnyei, 2007). The preliminary themes were further reviewed and refined through rereading the data, leading to the naming of the final themes. Main recursive themes include intentional knowledge acquiring, positive intercultural attitudes, and the transformation of cognition into actions.

## 4. Findings

### 4.1. Quantitative findings: The effects of the instructional design on students’ IC development

Cronbach Alpha testing was first conducted to check the internal reliability of each dimension of the ICS questionnaire. As shown in Table 4, the reliability of the knowledge dimension was 0.91 for the pretest and 0.93 for the post-test; the affective orientation dimension was 0.90 for the pretest and 0.87 for the post-test; the behavioral performance dimension was 0.92 for the pretest and 0.87 for the post-test; the cultural metacognition was.90 and.86 for the pretest and post-test, respectively.

The sample of 58 students had a mean score of 72.36 with a standard deviation of 12.74 for the overall questionnaire in the pretest. In the post-test, the mean score was 81.19 with a standard deviation of 12.02, displaying a change mean increase of 8.83. Table 4 further presents the mean score and standard deviation of each dimension of IC. It can be seen that the mean scores of students in the four IC dimensions improved to some extent after they participated in the instruction. To further examine whether these changes were meaningful before and after the instructional lesson, the paired sample t-test was conducted to compare the means of students’ performances in the pretest and post-test.

Table 5 shows the results of the paired sample t-test for the pretest and post-test of IC development in terms of the four dimensions. For students’ overall IC, there was a significant difference between the pretest and post-test (t = 4.92, *p* < 0.001). This could mean that the instructional design contributed effectively to students’ overall IC development. Concerning the dimension of IC knowledge, the t-value is 0.63 (*p* = 0.529 > 0.05). This indicates that there was no significant difference in students’ IC knowledge after receiving the instruction. It can be inferred that students did not perceive any progress in enhancing their cultural knowledge. Furthermore, the value of ps were less than.001 (*p* < 0.001) for the dimensions of affective orientation and cultural metacognition, suggesting that the instruction significantly facilitated students’ affective orientation and cultural metacognition. In the behavioral performance dimension, the value of p was.022 (*p* < 0.05). It can be assumed that the instruction was also significantly effective in enhancing students’ behavioral performance. From these quantitative findings, it can be concluded that the instructional design was effective in enhancing students’ IC in terms of affective orientation, behavioral performance, and cultural metacognition, but not in advancing their IC knowledge.

**Table 5:** Results of the paired-sample *t*-test (2-tailed).

		Paired Difference						
					95% Confidence Interval of the Difference			
		Mean	S.D.	Std. Error Mean	Lower	Upper	t	Sig. (2-tailed)
Pair 1	Post-test (knowledge) Pretest (knowledge)	0.62	7.46	0.98	−1.34	2.58	0.63	0.529
Pair 2	Post-test(affective orientation) Pretest (affective orientation)	3.02	5.85	0.77	1.48	4.56	3.93	< 0.001***
Pair 3	Post-test (behavioral performance) Pretest (behavioral performance)	1.78	5.72	0.75	0.271	3.28	2.36	0.022**
Pair 4	Post-test (cultural metacognition) Pretest (cultural metacognition)	3.41	4.83	0.63	2.15	4.68	5.39	< 0.001***
Pair 5	Post-test (intercultural competence) Pretest (intercultural competence)	8.83	13.67	1.79	5.23	12.42	4.92	< 0.001***

### 4.2. Qualitative findings: How cultural metacognition facilitates students’ IC development

By coding and analyzing the qualitative data gained from focus group interviews, general themes around the effects of the instructional design on students’ IC development emerged. The main themes are synthesized as the following to answer the second research question.

#### 4.2.1. Support intentional knowledge acquiring

Most students brought forward that the guiding questions in the Autobiography task prompted them to continuously check their knowledge base during the process. At the same time, they showed a strong willingness to gain knowledge through self-learning. The following excerpts manifest students’ desire and readiness to acquire new cultural knowledge driven by the metacognitive teaching techniques.

Answering the guiding questions was a tough process, but the questions kept me wondering if I had the right understanding of their background. Therefore, I had to do some research on the Internet to fill my knowledge gaps.

In the group discussion, the questions motivated us to reflect on what we know and what we don’t know. After each presentation, the reflective writing also made us reflect on the weaknesses of our knowledge and what other aspects of knowledge should we improve on.

Some students also reported that they benefited from the explicitly taught metacognitive strategies. For example, Xiao started to reexamine the cultural knowledge she possessed when she deliberately used the perspective-taking strategy.

The teacher taught us how to put ourselves in other people's shoes. This made me realize that I didn’t have a concrete concept of their culture before. But we have sought some information for us to get closer to Malaysian culture.

This concurs with Liao and Thomas (2020) that cultural metacognition regulates one’s attention on the cultural knowledge and controls one’s cognitive processing in order to achieve one’s goals. When learners deliberately monitor and reflect on their cultural knowledge use in intercultural encounters, they focus on managing knowledge relevant to intercultural interactions. Accordingly, they tend to intentionally acquire new cultural knowledge relevant to certain intercultural tasks.

#### 4.2.2. Develop positive intercultural attitudes

The qualitative data show that students developed positive attitudes regarding other cultures, including curiosity, respect, openness, tolerance, overcoming stereotypes and prejudice, and withholding judgment. The following excerpt demonstrates how one student (Zhang) first saw her culture as the norm and neglected the impact of different cultures on people’s experiences. Then, by reflecting on her own thoughts, Zhang realized her stereotypes and adopted an open mind and a willingness to understand and respect other cultures.

Before this, I had thought people should wear formal clothing on important occasions. In our culture, flip-flops should not be worn on formal occasions. When we were reflecting on what led to the difference between us and them, I realized that it was our stereotypes that led to this view. They (Myanmar leaders) wear slippers in the same sense that our leaders wear Zhongshan suits and traditional Chinese costumes for formal occasions, and in the same way that people in some Western countries wear suits with ties and top hats in formal settings. We should respect the culture of other countries and open our minds. We can't simply make judgments until we learn about them.

It can be seen that driven by the reflective questions, Zhang tried to evaluate and examine her primary thoughts by empathizing with other cultures. Empathy, a key component of cultural metacognition, helps individuals to deal with stereotypes, prejudices, and ethnocentrism (Arasaratnam, 2013; Wang and Kulich, 2015). Zhang effectively used empathy to shift from her original ethnocentric view, which takes her own cultural perspective as the ideal norm for viewing other cultures, to an ethnorelative view, which adopts other perspectives to accommodate the values of other cultures. During this process, it was her metacognitive thinking facilitated her efforts to attenuate the influence of her primary thoughts on her judgment and attitudes. This is consonant with the views proposed by Petty et al. (2007) and Wegener et al. (2012). As a secondary thought, metacognition can help individuals search for negative or inappropriate thoughts, and prompt them to regulate previous thoughts and attitudes. Viewed from this angle, cultural metacognition helps learners to overcome stereotypes and develop open attitudes toward other cultures.

#### 4.2.3. Promote the transformation of cognition into action

Thomas (2006), Van der Horst and Albertyn (2018) and Liao and Thomas (2020) propose that cultural metacognition regulates cognition and provides a link between cognition and appropriate behaviors during intercultural interactions. Cognition includes knowledge of cultural norms, practices, and conventions gained through educational or personal experiences (Ang and Van Dyne, 2008). As behaviors are enacted largely in a conscious way, cognition and metacognition provide a basis for behaviors (Earley and Peterson, 2004; Sharma, 2019). However, little empirical attempt has been made to explore the mechanism of how cultural metacognition help to build this link. In this study, the qualitative data provide a glimpse into this. The following excerpt demonstrates that one student (Ye) with high cultural metacognition adapted her behavioral and communicative strategies in the role-play so that she could act more culturally appropriately. It can be seen that Ye went through metacognitive processes including being continuously and proactively aware of her cultural cognition, carefully controlling her ethnocentric thoughts, managing knowledge related to intercultural interactions, and deliberately planning appropriate behaviors prior to the interaction.

I always reminded myself that I should not use my own cultural values to judge others’ cultural habits, let alone demand others to follow our cultural standards. Therefore, I first learned the simple greetings of the Hui people and imitated their unique ways of greeting. In our role-play of the dining scene, I avoided topics like eating pork, drinking, or smoking. Do in Rome as Rome does, and respect their culture.

Another student, Chen, reported on her self-regulation of cognition in order to achieve desired outcomes, which was to receive the client appropriately. Her group chose an image showing a man from West Africa with his two wives. Although Chen had her own judgment toward the people shown in the image, she still chose to inhibit undesirable behaviors as a result of her active monitoring and controlling of her cognitive processes. Her purposeful metacognitive thoughts facilitated her to focus more on the motives and goals of the interaction, which further prompted her to avoid inappropriate or undesired behaviors.

When I first saw the image, I could only say that our cultures are so different. In China, polygamy is illegal. Also, as a woman, I felt it was unacceptable because women are not respected in such marriages. However, if I had to meet someone from this culture as my client, I would not bring up a topic related to this because I don’t think he or she would expect or welcome judgments on their cultural habits.

## 5. Discussion and implications

### 5.1. Benefits of the instructional design featuring cultural metacognition in EFL context

Quantitative findings provide evidence for the first research question that the instructional design featuring cultural metacognition was effective in facilitating participants’ IC development. The 58 participants significantly showed development in their self-perceived IC in terms of affective orientation, behavioral performance, and cultural metacognition as measured by the ICS questionnaire, but they did not perceive significant growth in their intercultural knowledge. Furthermore, as confirmed by qualitative results, participants showed an increased level of positive attitudes and behavioral performance along with their improved cultural metacognitive thinking through participation in the instruction. In general, these different dimensions of IC development can be explained by the process-oriented model of IC proposed by Deardorff (2006), which emphasizes the cyclical development of IC with the facilitation of all components. Accordingly, the increased cultural metacognition may contribute to enhanced affective orientation and behavioral performance.

As for the knowledge dimension, qualitative data suggest that participants were willing and ready to acquire new cultural knowledge driven by the reflective questions and the explicitly taught metacognitive strategies. However, consonant with the quantitative findings, there was no evidence to support participants’ increased knowledge. This may be due to two reasons. On the one hand, the instructional activities only involved students focusing on specific cultural knowledge relevant to particular images. They did not get the chance to further enrich culture-general and culture-hybrid knowledge as reflected in the questionnaire, such as common rules and values of other cultures, and knowledge about the negotiation strategies. On the other hand, when students constantly monitor their knowledge use with the aid of cultural metacognition strategies, they may feel that their knowledge is still insufficient for them to handle all sorts of intercultural interactions. As a result, students with such concerns may not show significant improvement in their self-rated performance of intercultural knowledge.

To answer the second research question of “how does cultural metacognition contribute to students’ IC development?,” three general themes in relation to the cognitive, affective, and behavioral dimensions emerged from qualitative data, namely supporting intentional knowledge acquiring, developing positive intercultural attitudes, and promoting the translation of cognition into actions. The first two findings concur with previous studies (e.g., Petty et al., 2007; Wegener et al., 2012; Liao and Thomas, 2020) that cultural metacognition provides an impetus for learners to manage cognition in order to complete certain intercultural tasks and develop positive intercultural attitudes toward novel intercultural situations. The current study further confirms the effectiveness of teaching techniques focusing on cultural metacognition, such as reflective questions and written tasks, and explicitly taught cultural metacognitive skills including self-monitoring, perspective-taking, and mindfulness. Moreover, it is noteworthy that students attempted to improve their cognition relevant to intercultural interactions through active self-learning, which is an indispensable asset both for IC development and lifelong learning. Shifting attention to the behavioral aspect of IC, although prior studies (Thomas, 2006; Van der Horst and Albertyn, 2018; Liao and Thomas, 2020)) have theoretically proposed the critical role of cultural metacognition in linking cognition and culturally-appropriate behavior, this study provides empirical evidence that cultural metacognition functions through continuous proactive awareness, monitoring and controlling of one’s cognition, focusing on knowledge relevant to the intercultural interaction, and intentional planning of appropriate behaviors before the interaction.

### 5.2. Implications for IC teaching in EFL context

The current study not only confirms the effectiveness of the instructional design featuring cultural metacognition in enhancing different dimensions of IC in EFL context, but also offers additional evidence of how this was achieved through a range of metacognitive processes. Accordingly, it provides two implications for practical IC teaching in EFL context.

First, this study provides evidence for the value of teaching techniques, such as explicit instructions on metacognitive skills and reflective practices, for promoting learners’ cultural metacognition and further leading to IC development. As mentioned earlier, while these techniques have been verified as efficient ways of promoting metacognitive skills (e.g., Bae and Kwon, 2019; O’Loughlin and Griffith, 2020; Santangelo et al., 2021), there are few empirical studies offer insights into how could these techniques contribute to cultural metacognition development in the context of intercultural education. Earley and Peterson (2004) suggest that a specific discussion of metacognitive strategies in intercultural training could promote learners’ ability to transfer a concept from an example case to a novel situation, and this study further enriches and extends the positive effects of cultural metacognition on EFL learners’ IC development. Furthermore, this study suggests that these teaching techniques should be incorporated into IC teaching routines rather than a one-time training so that students could develop habits of monitoring, regulating, planning, and reflecting and use these metacognitive skills more often in new intercultural contexts. Moreover, as IC development is an ongoing and cyclical process (Deardorff, 2006), teaching activities should not only focus on cultural metacognition but also on other IC components in order to develop learners’ IC comprehensively.

Second, as previously stated, one of the key challenges of developing EFL learners’ IC in Chinese domestic higher education context is the lack of authentic intercultural experiences. Deardorff (2012) also argues that IC development does not occur in a vacuum, and as indicated by his IC model, it is through interactions that individuals turn internal metacognitive outcomes into external behavioral outcomes. Considering this deficiency of implementing IC teaching in EFL context, this study employed group work to encourage students to interact with others who may hold different cultural values in the Autobiography task, and it employed role-play to simulate intercultural interactions. Although these interactions were not enough to encompass the complexities and uncertainties of authentic intercultural encounters, cultural metacognition could equip students with the necessary skills to face what they will encounter in new situations. IC development not only occurs in interactions with people of highly diverse cultural backgrounds, but also happens in classroom processes, and in interactions between co-cultural groups of students and between teachers and students (Wang and Kulich, 2015). What is critical is preparing learners with higher-order and transferable skills. Thus, this calls for morew practical teaching and research to explore the role of cultural metacognition in IC learning in EFL contexts.

## 6. Conclusion

This study argues the crucial role of cultural metacognition in intercultural learning and examines whether and how an instructional design specifically integrated cultural metacognition could contribute to learners’ IC development in EFL classrooms. The findings suggest that the instructional design facilitated learners’ IC development in terms of affective, metacognitive, and behavioral dimensions. In addition, this study did not find a significant change in students’ IC knowledge. This study also provides evidence that the teaching techniques focusing on cultural metacognition, such as explicit coaching and discussion on metacognitive skills, and reflective questions and writing, could support students’ intentional knowledge acquiring, help students develop positive intercultural attitudes, and promote the translation of cognition into actions. These are exciting and important contributions as they could potentially lead to the cyclical development of IC. Insights gained from this study could provide teachers with practical techniques and suggestions for implementing IC instructions in EFL contexts.

While efforts were made to ensure the study was conducted meticulously, there are some limitations that could hinder its generalizability and contributions. First and foremost is the sample size which is relatively small. This inhibits generalization beyond the sampled course and students. Replication can be conducted with a larger sample of students. In addition, more long-term and routinely-structured IC teaching with an emphasis on cultural metacognition are encouraged in order to further validate the effects of cultural metacognition on students’ IC development. Furthermore, as only one group of students was selected as participants in this study, a control group of students who do not participate could be included in ordeFr to check if there are other factors that may also affect students’ IC development. Last, as both quantitative and qualitative data collected were students’ perceptions which might be subjective, this study was not adequately designed to assess how much the instructional design promoted students’ IC. Future research may explore other assessment measures to provide a more comprehensive and objective understanding of learners’ IC development.

## Data availability statement

The original contributions presented in the study are included in the article/supplementary material, further inquiries can be directed to the corresponding author.

## Ethics statement

The studies involving human participants were reviewed and approved by the Research Ethics Committee of School of Foreign Languages, Xinyang Agriculture and Forestry University. The patients/participants provided their written informed consent to participate in this study.

## Author contributions

LH was in charge of conception, design, research execution, data collection and analysis, drafting and revising of manuscript, and funding acquisition.

## Funding

This research was supported and funded by Xinyang Agriculture and Forestry University Young Teacher Research Fund (QN2022047).

## Conflict of interest

The author declares that the research was conducted in the absence of any commercial or financial relationships that could be construed as a potential conflict of interest.

## Publisher’s note

All claims expressed in this article are solely those of the authors and do not necessarily represent those of their affiliated organizations, or those of the publisher, the editors and the reviewers. Any product that may be evaluated in this article, or claim that may be made by its manufacturer, is not guaranteed or endorsed by the publisher.

## References

[ref1] Ang S. Van Dyne L. (2008). "Conceptualization of cultural intelligence: definition, distinctiveness, and nomological network," in Handbook of Cultural Intelligence*.,* eds. Ang S. Dyne L. Van. (New York: Sharpe), 3–15.

[ref2] Ang S. Van Dyne L. Koh C. (2006). Personality correlates of the four-factor model of cultural intelligence. Group Org. Manag. 31, 100–123. doi: 10.1177/1059601105275267

[ref3] Ang S. Van Dyne L. Rockstuhl T. (2015). “Cultural intelligence: origins, conceptualization, evolution, and methodological diversity” in Handbook of Advances in Culture and Psychology. eds. Gelf M. J. Chiu C.-Y. Hong Y.-Y. (Oxford: Oxford University Press), 273–323.

[ref4] Arasaratnam L. A. (2013). A review of articles on multiculturalism in 35 years of IJIR. Int. J. Intercult. Relat. 37, 676–685. doi: 10.1016/j.ijintrel.2013.09.006

[ref5] Bae H. Kwon K. (2019). Developing metacognitive skills through class activities: what makes students use metacognitive skills? Educ. Stud. 47, 456–471. doi: 10.1080/03055698.2019.1707068

[ref6] Bannert M. Sonnenberg C. Mengelkamp C. Pieger E. (2015). Short-and long-term effects of students’ self-directed metacognitive prompts on navigation behavior and learning performance. Comput. Hum. Behav. 52, 293–306. doi: 10.1016/j.chb.2015.05.038

[ref7] Barrett M. (2018). How schools can promote the intercultural competence of young people. Eur. Psychol. 23, 93–104. doi: 10.1027/1016-9040/a000308

[ref8] Bennett M. J. (1998). “Intercultural communication: a current perspective” in Basic Concepts of Intercultural Communication: Selected Readings. ed. Benette M. J. (Yarmouth, ME: Intercultural Press)

[ref9] Bird A. Mendenhall M. Stevens M. J. Oddou G. (2010). Defining the content domain of intercultural competence for global leaders. J. Manag. Psychol. 25, 810–828. doi: 10.1108/02683941011089107

[ref10] Bogilović S. Černe M. Škerlavaj M. (2017). Hiding behind a mask? Cultural intelligence, knowledge hiding, and individual and team creativity. Eur. J. Work Organ. Psy. 26, 710–723. doi: 10.1080/1359432X.2017.1337747

[ref11] Bransford J. Brown A. Cocking R. (1999). How People Learn: Brain, Mind, Experience, and School. Washington, DC: National Research Council.

[ref12] Braun V. Clarke V. (2006). Using thematic analysis in psychology. Qual. Res. Psychol. 3, 77–101. doi: 10.1191/1478088706qp063oa

[ref13] Brislin R. Worthley R. Macnab B. (2006). Cultural intelligence: understanding behaviors that serve people’s goals. Group Org. Manag. 31, 40–55. doi: 10.1177/10596011052752

[ref14] Bryce D. Whitebread D. Szűcs D. (2015). The relationships among executive functions, metacognitive skills and educational achievement in 5 and 7 year-old children. Metacogn. Learn. 10, 181–198. doi: 10.1007/s11409-014-9120-4

[ref15] Byram M. (1997). Teaching and Assessing Intercultural Communicative Competence. Bristol: Multilingual Matters.

[ref16] Byram M. Barrett M. Ipgrave J. Jackson R. Méndez García M. C. (2009). Autobiography of Intercultural Encounters. Strasbourg: Council of Europe Publishing.

[ref17] Chao T.-C. (2014). The development and application of an intercultural competence scale for university EFL learners. 英語教學期刊 38, 79–124. doi: 10.6330/ETL.2014.38.4.04

[ref18] Chin T. Meng J. Wang S. Shi Y. Zhang J. (2022). Cross-cultural metacognition as a prior for humanitarian knowledge: when cultures collide in global health emergencies. J. Knowl. Manag. 26, 88–101. doi: 10.1108/JKM-10-2020-0787

[ref19] Chiu C.-Y. Lonner W. J. Matsumoto D. Ward C. (2013). Cross-cultural competence: theory, research, and application. J. Cross-Cult. Psychol. 44, 843–848. doi: 10.1177/0022022113493716

[ref20] Chua R. Y. Morris M. W. Mor S. (2012). Collaborating across cultures: cultural metacognition and affect-based trust in creative collaboration. Organ. Behav. Hum. Decis. Process. 118, 116–131. doi: 10.1016/j.obhdp.2012.03.009

[ref21] Chua R. Y. Ng K. Y. (2017). Not just how much you know: interactional effect of cultural knowledge and metacognition on creativity in a global context. Manag. Organ. Rev. 13, 281–300. doi: 10.1017/mor.2016.32

[ref22] Cohen L. Manion L. Morrison K. (2018). Research Methods in Education (8th). New York: Routledge.

[ref23] Deardorff D. K. (2006). Identification and assessment of intercultural competence as a student outcome of internationalization. J. Stud. Int. Educ. 10, 241–266. doi: 10.1177/1028315306287002

[ref24] Deardorff D. K. (2012). Framework: Intercultural competence model, in Building Cultural Competence: Innovative Activities and Models, eds. Berardo K. Deardorff D. K. (Sterling: Stylus Publishing), 45–52.

[ref25] Deardorff D. K. (2015). Demystifying Outcomes Assessment for International Educators: A Practical Approach. Sterling: Stylus Publishing, LLC.

[ref26] Dörnyei Z. (2007). Research Methods in Applied Linguistics. Oxford: Oxford University Press.

[ref27] Dypedahl M. (2018). “A metacognitive approach to intercultural learning in language teacher education” in Metacognition in Language Learning and Teaching. eds. Haukås Å. Bjørke C. Dypedahl M. (New York: Routledge), 48–66.

[ref28] Earley P. C. Ang S. Tan J.-S. (2006). CQ: Developing Cultural Intelligence at Work. Redwood City: Stanford University Press.

[ref29] Earley P. C. Peterson R. S. (2004). The elusive cultural chameleon: cultural intelligence as a new approach to intercultural training for the global manager. Acad. Manag. Learn. Edu. 3, 100–115. doi: 10.5465/AMLE.2004.12436826

[ref001] Fang F. Baker W. (2018). A more inclusive mind towards the world’: English language teaching and study abroad in China from intercultural citizenship and English as a lingua franca perspectives. Lang. Teach. Res. 22, 608–624.

[ref30] Fantini A. E. (2009). “Assessing intercultural competence” in The SAGE Handbook of Intercultural Competence. ed. Deardorff D. K. (Thousand Oaks: SAGE Publications), 456–476. doi: 10.4135/9781071872987.n27

[ref31] Feng A. (2009). “Becoming interculturally competent in a third space” in Becoming Interculturally Competent through Education and Training. ed. Fleming M. P. (Bristol: Multilingual Matters), 71–91. doi: 10.21832/9781847691644-008

[ref32] Flavell J. H. (1987). “Speculations about the nature and development of metacognition” in Metacognition, Motivation and Understanding. eds. Weinert F. Kluwe R. (Hillsdale: Erlbaum), 21–29.

[ref33] Griffith R. L. Wolfeld L. Armon B. K. Rios J. Liu O. L. (2016). Assessing intercultural competence in higher education: existing research and future directions. ETS Res. Rep. Ser. 2016, 1–44. doi: 10.1002/ets2.12112

[ref34] Gu X. Zhao Y. (2021). Integration of teaching and assessing intercultural communicative competence. Chin. J. Appl. Linguist. 44, 241–258. doi: 10.1515/CJAL-2021-0014

[ref003] Hacker D. J. Dunlosky J. Graesser A. C. (1998). Metacognition in educational theory and practice. New York: Routledge

[ref35] Huang L.-J. D. (2021). Developing intercultural communicative competence in foreign language classrooms–a study of EFL learners in Taiwan. Int. J. Intercult. Relat. 83, 55–66. doi: 10.1016/j.ijintrel.2021.04.015

[ref36] Klafehn J. Banerjee P., and Chiu, C.-y. (2008). Navigating cultures: a model of metacognitive intercultural intelligence, in Handbook of Cultural Intelligence, eds. Ang S. Van Dyne L. (New York: Sharpe), 318–331.

[ref37] Kramarski B. Mevarech Z. R. (2003). Enhancing mathematical reasoning in the classroom: the effects of cooperative learning and metacognitive training. Am. Educ. Res. J. 40, 281–310. doi: 10.3102/00028312040001281

[ref38] Lane H. C. (2009). “Promoting metacognition in immersive cultural learning environments” in Human-Computer Interaction. ed. Jacko J. A. (Berlin: Springer)

[ref39] Lee S. Adair W. L. Seo S.-J. (2013). Cultural perspective taking in cross-cultural negotiation. Group Decis. Negot. 22, 389–405. doi: 10.1007/s10726-011-9272-4

[ref40] Leung A. K.-Y. Lee S.-I. Chiu C.-Y. (2013). Meta-knowledge of culture promotes cultural competence. J. Cross-Cult. Psychol. 44, 992–1006. doi: 10.1177/002202211349313

[ref41] Li M. (2020). An examination of two major constructs of cross-cultural competence: cultural intelligence and intercultural competence. Personal. Individ. Differ. 164:110105. doi: 10.1016/j.paid.2020.110105

[ref42] Liao Y. Thomas D. C. (2020). Cultural Intelligence in the World of Work. Berlin: Springer.

[ref43] Loewenstein J. Thompson L. Gentner D. (2003). Analogical learning in negotiation teams: comparing cases promotes learning and transfer. Acad. Manag. Learn. Edu. 2, 119–127. doi: 10.5465/AMLE.2003.9901663

[ref44] Malek M. A. Budhwar P. (2013). Cultural intelligence as a predictor of expatriate adjustment and performance in Malaysia. J. World Bus. 48, 222–231. doi: 10.1016/j.jwb.2012.07.006

[ref45] McConachy T. Liddicoat A. J. (2016). “Meta-pragmatic awareness and intercultural competence: the role of reflection and interpretation in intercultural mediation” in Intercultural Competence in Education. eds. Dervin F. Gross Z. (London: Palgrave Macmillan), 13–30.

[ref46] Metcalfe J. Finn B. (2008). Evidence that judgments of learning are causally related to study choice. Psychon. Bull. Rev. 15, 174–179. doi: 10.3758/PBR.15.1.174, PMID: 18605499

[ref002] Mor S. Morris M. W. Joh J. (2013). Identifying and training adaptive cross-cultural management skills: The crucial role of cultural metacognition. Acad. Manag. Learn. Educ 12, 453–475.

[ref47] Morris M. W. Savani K. Fincher K. (2019). Metacognition fosters cultural learning: evidence from individual differences and situational prompts. J. Pers. Soc. Psychol. 116, 46–68. doi: 10.1037/pspi0000149, PMID: 30596446

[ref004] National Bureau of Statistics (2021). 第七次全国人口普查剬报 [The seventh national census].[Online]. Available at: http://www.gov.cn/guoqing/2021-05/13/content_5606149.htm (Accessed December 12, 2022).

[ref48] Nunaki J. Damopolli I. Kandowangko N. Nusantri E. (2019). The effectiveness of inquiry-based learning to train the students' metacognitive skills based on gender differences. Int. J. Instr. 12, 505–516. doi: 10.29333/iji.2019.12232a

[ref49] O’Loughlin V. D. Griffith L. M. (2020). Developing student metacognition through reflective writing in an upper level undergraduate anatomy course. Anat. Sci. Educ. 13, 680–693. doi: 10.1002/ase.1945, PMID: 31965753

[ref50] Ogan A. Aleven V. Jones C. (2009). Advancing development of intercultural competence through supporting predictions in narrative video. Int. J. Artif. Intell. Educ. 19, 267–288.

[ref51] Peng A. C. Van Dyne L. Oh K. (2015). The influence of motivational cultural intelligence on cultural effectiveness based on study abroad: the moderating role of participant’s cultural identity. J. Manag. Educ. 39, 572–596. doi: 10.1177/10525629145557

[ref52] Petty R. E. Briñol P. Tormala Z. L. Wegener D. T. (2007). “The role of metacognition in social judgment” in Social Psychology: A Handbook of Basic Principles. eds. Higgins E. T. Kruglanski A. W. 2nd ed (New York: Guilford Press), 254–284.

[ref53] Phelan J. E. (2019). Teaching and learning cultural metacognition in marketing and sales education. Int. J. Market. Sales Educ. 2, 18–29. doi: 10.4018/IJMSE.2019070102

[ref54] Rockstuhl T. Van Dyne L. (2018). A bi-factor theory of the four-factor model of cultural intelligence: meta-analysis and theoretical extensions. Organ. Behav. Hum. Decis. Process. 148, 124–144. doi: 10.1016/j.obhdp.2018.07.005

[ref55] Rodríguez L. M. G. Carranza T. R. (2017). “Promoting intercultural competence through cross-cultural projects and literature” in Proceedings of the International Colloquium on Languages, Cultures, Identity, in School and Society. ed. Ramos F., (Los Angeles: School of Education at Digital Commons) 79–85.

[ref56] Ruben B. D. (1989). The study of cross-cultural competence: traditions and contemporary issues. Int. J. Intercult. Relat. 13, 229–240. doi: 10.1016/0147-1767(89)90011-4

[ref57] Santangelo J. Cadieux M. Zapata S. (2021). Developing student metacognitive skills using active learning with embedded metacognition instruction. J. STEM Educ. Innov. Res. 22, 51–63.

[ref58] Sercu L. (2004). Assessing intercultural competence: a framework for systematic test development in foreign language education and beyond. Intercult. Educ. 15, 73–89. doi: 10.1080/1467598042000190004

[ref59] Sharma R. R. (2019). Cultural intelligence and institutional success: the mediating role of relationship quality. J. Int. Manag. 25:100665. doi: 10.1016/j.intman.2019.01.002

[ref60] Spitzberg B. H. Changnon G. (2009). “Conceptualizing intercultural competence” in The Sage Handbook of Intercultural Competence. ed. Deardorff D. K. (Thousand Oaks: SAGE Publications), 2–52.

[ref61] Tan J. S. Chua R. Y. J. (2003). “Training and developing cultural intelligence” in Cultural Intelligence: An Analysis of Individual Interactions across Cultures. eds. Earley P. C. Ang S. (Palo Alto: Stanford University Press), 258–303.

[ref62] Thomas D. C. (2006). Domain and development of cultural intelligence: the importance of mindfulness. Group Org. Manag. 31, 78–99. doi: 10.1177/1059601105275266

[ref63] Triandis H. C. (2006). Cultural intelligence in organizations. Group Org. Manag. 31, 20–26. doi: 10.1177/1059601105275253

[ref64] Van der Horst C. A. Albertyn R. M. (2018). The importance of metacognition and the experiential learning process within a cultural intelligence–based approach to cross-cultural coaching. SA J. Hum. Resour. Manag. 16, 1–11. doi: 10.4102/sajhrm.v16i0.951

[ref65] Van Dyne L. Ang S. Ng K. Y. Rockstuhl T. Tan M. L. Koh C. (2012). Sub-dimensions of the four factor model of cultural intelligence: expanding the conceptualization and measurement of cultural intelligence. Soc. Personal. Psychol. Compass 6, 295–313. doi: 10.1111/j.1751-9004.2012.00429.x

[ref66] Wang Y. A. Kulich S. J. (2015). Does context count? Developing and assessing intercultural competence through an interview-and model-based domestic course design in China. Int. J. Intercult. Relat. 48, 38–57. doi: 10.1016/j.ijintrel.2015.03.013

[ref67] Wang M.-J. Yang L.-Z. Chen T.-I. (2020). The effectiveness of ICT-enhanced learning on raising intercultural competencies and class interaction in a hospitality course. Interact. Learn. Environ. 28, 1–13. doi: 10.1080/10494820.2020.1815223

[ref005] Wegener D. T. Silva P. P. Petty R. E. Garcia-Marques T. (2012). “The metacognition of bias regulation” in Social metacognition. eds. Briñol P. DeMarree K. G. (New York: Psychology Press), 81–99.

[ref68] Yzerbyt V. Y. Demoulin S. (2012). “Metacognition in stereotypes and prejudice” in Social Metacognition. eds. Briñol P. DeMarree K. G. (New York: Taylor & Francis), 243–262.

[ref69] Zhang X. Zhou M. (2019). Interventions to promote learners’ intercultural competence: a meta-analysis. Int. J. Intercult. Relat. 71, 31–47. doi: 10.1016/j.ijintrel.2019.04.006

[ref70] Zhao H. Coombs S. (2011). Intercultural teaching and learning strategies for global citizens: a Chinese EFL perspective. Teach. High. Educ. 17, 245–255. doi: 10.1080/13562517.2011.611874

